# Sperm biology and male reproductive health

**DOI:** 10.1038/s41598-020-78861-7

**Published:** 2020-12-14

**Authors:** Ricardo P. Bertolla

**Affiliations:** Division of Urology, Department of Surgery, Universidade Federal de São Paulo, Rua Napoleão de Barros, 715, 2º andar, São Paulo, SP 04024-002 Brazil

**Keywords:** Urology, Cell biology

## Abstract

Sperm are unique cells, produced through the complex and precisely orchestrated process of spermatogenesis, in which there are a number of checkpoints in place to guarantee delivery of a high-quality and high-fidelity DNA product. On the other hand, reproductive pressure in males means that to produce more is, in very general terms, to perform better. Balancing quantity and quality in sperm production is thus a delicate process, subject to specific cellular and molecular control mechanisms, and sensitive to environmental conditions, that can impact fertility and offspring health. This Collection is focused on these aspects of sperm biology, as well as their impact on reproductive performance and male infertility.

Anisogamy, defined as gamete size dimorphism alongside binary fusion, is the most observed gametic system in multicellular organisms^1^. Early mathematical anisogamy models demonstrated that the most efficient rate of gamete fusion would be achieved if small and large gametes were produced, due to increased collision events (reviewed by Lehtonen and Parker^2^). Under this understanding, males may be generically defined as those counterparts that produce smaller gametes (i.e., sperm), while females conversely produce the larger ones (ova)^3^. Both gametes, thus, have accumulated very specialized traits in terms of cellular morphological specialization (and polarity, in sperm), molecular machinery, and responsiveness to external stimuli.

Sperm are unique in that they carry out their functions outside their organism of origin. In humans, for example, fertilization occurs within the uterine tubes, in the female reproductive tract^4^. In some species of fish, fertilization occurs under different osmotic pressures^5^. During their development, sperm therefore need to be prepared, as best as possible, for all the possible hindrances and events that will occur between the moment they are released and the moment they effectively penetrate the oocyte. One could putatively divide the life of each sperm into four general moments: (i) spermatogenesis—the process by which diploid spermatogonia undergo meiosis and morphological alterations to produce sperm; (ii) post testicular maturation and storage—the moment after spermatogenesis but before ejaculation, in which sperm interact with extracellular proteins, lipids and microRNAs, among other molecules; (iii) capacitation—the moment after ejaculation in which sperm effectively acquire the capacity to fertilize and oocyte; and (iv) fertilization—the process by which the spermatozoon penetrates the oocyte’s outer protective layers and transfers its genomic material to the oolemma, the initial step in the formation of a zygote (fully reviewed in^6^).

Spermatogenesis is directly connected to testicular physiology. This is initially determined by testis formation during fetal development. In humans, use of endocrine disruptors by pregnant women, for example, has been shown to be associated with future infertility in their male offspring^7^. Holland et al. demonstrated that there is no effect of a maternal androgenic environment (due to polycystic ovarian syndrome) on the male reproductive axis^8^, while Lessard et al. demonstrated that prenatal exposure to persistent organic pollutants reduced semen quality in male offspring^9^. After the onset of puberty, with the development of secondary sexual traits, spermatogenesis initiates, during which there are a number of conditions that may affect the quality of ejaculates—varicocele, for example, is detected in up to 80% of men with secondary infertility^10^, while testicular heat stress, in bovines, is a cause for lower fertility rates in sires^11^.

In a recent analysis of a large retrospective cohort, Siqueira et al. observed that, over the previous 23 years, semen quality has been steadily decreasing in men attending a reference infertility center in Brazil^12^. This fairly large cohort (a little over 9000 men) agrees, as the authors discussed, with other studies that have observed a downward trend in semen quality over the past decades. There is still much debate as to the reasons for this trend, ranging from artifactual alteration due to subjectivity in some of the analyses, to increased awareness leading to more men with potential fertility problems seeking evaluation, to a true decrease in male reproductive health. Whichever the case, the authors demonstrated that the decrease in semen quality is observed even when only an objective measure, such as total motile sperm count, is utilized.

This has led to the obvious association between environmental conditions and testicular alterations. Greeson et al. examined environmental exposure to PBB153, a polybrominated biphenyl compound used as a flame retardant that accidentally entered the food chain in 1973, and led to alterations in liver and thyroid functions, as well as to increased odds of lymphomas and cancers of the digestive tract. The authors demonstrated that higher serum PBB153 levels were associated with decreased methylation of imprint control regions, which in turn demonstrated that this contaminant determines alterations to the germ line which are then potentially transferred to the offspring of these men^13^. Other environmental effects, such as diets, are more difficult to measure. While some studies have observed effects of obesity on male fertility, others have not. Gómez-Elías et al. studied the effect of high fat diets in mice on high reproductive performance and did not observe a detrimental effect^14^. Elenkov et al., on the other hand, did observe that men who were treated by ICSI had a higher risk for being under treatment for hypertension or metabolic syndrome^15^. Interestingly, a mechanistic cause for hypertension, as it leads to testicular alteration, was later demonstrated by Colli et al.^16^.

The next point of interest, after understanding how the environment affects the testes, would be to understand how sperm interact with their environment. Epididymal proteins and RNAs actively interact with sperm via extracellular vesicles in order to modulate sperm activity^17^. Increased epididymal expression of interleukin 6, for example, has been shown to lead to male infertility in a mouse model^18^. Extracellular vesicle communication remains important after ejaculation. Studies have shown that, in humans, prostatic fluid releases prostasomes that modulate acrosome reaction^17^. Murdica et al. demonstrated that endometrial cells also produce extracellular vesicles that are uptaken by sperm^19^. This is an important demonstration that sperm remain responsive to environmental conditions well after ejaculation, and that this is likely a means by which they acquire the capacity to adapt to the female reproductive tract. If this also holds true for species that present external fertilization remains to be demonstrated.

While this Collection is dedicated to observing this and many other questions, I believe the articles generally fall within the four putative steps presented at the initial portion of this text, with the caveat that biotechnologies may affect one or more of these steps at any given time. Given the broad spectrum that sperm biology covers, both in terms of species and in terms of the many events that are associated, this Collection has sought to bring together as much as possible, while stringing these together under three general areas of research: (i) testis, sperm, and their environment; (ii) biotechnologies and assisted reproduction; and (iii) clinical, experimental, and epidemiologic studies on male infertility.

In sexual reproduction, life begins with the fusion of two gametes. This is the best answer one can produce to the age-old question of where we come from. To understand the life and times of sperm is to understand the foundations of our biological origins, and is a gateway towards understanding early development.
